# Strategies for improvement of WeChat-PBL teaching: experience from China

**DOI:** 10.5116/ijme.582e.015a

**Published:** 2016-11-28

**Authors:** Furong Zeng, Guangtong Deng, Zhao Wang, Shi Chang, Xiang Chen, Lin Qi, Xiongbing Zu, Longfei Liu

**Affiliations:** 1 Xiangya Medical School ,Central South University, China; 2 Xiangya Hospital, Central South University, China

**Keywords:** WeChat, PBL, teaching, China

## Introduction

Problem-based learning (PBL) is an approach to learning that is used to a greater degree in many medical schools worldwide. It surpasses traditional teaching not only in fostering understanding and the retention of knowledge,^1^ but also in developing social and cognitive abilities.^2^ However, it has limitations including more time consumption,^3^ concerns about overlooking things, the high self-motivation required and a predominating uncertainty about the breadth and depth of learning without a syllabus.^4^ How to avoid these shortcomings interests many medical workers. Not long ago, we have put forward WeChat-PBL teaching, which has proved efficient in overcoming its disadvantages.^5^ Now, we suggest some tips to exert its best benefits in WeChat-PBL teaching.

### Preparation of the faculty

Traditional teaching model has a deep-rooted influence on a majority of faculty, so one of the essential steps in the process of curriculum change is to prepare the faculty. First, the teaching faculty members should be given lessons to understand the changes and the reasons for these changes before they teach the class. Second, the university should give a lecture about the operating of WeChat-PBL system. Feedback on the proposal should be provided to deal with faculty questions, and handle any fears or uncertainty anytime during the teaching process. When faculty members become aware about their roles in the new PBL curriculum and what they need to do, they usually perform better.^6^ Additionally, the universities can provide an unique teaching award to encourage a reflection on teaching, validating techniques, increase their passion for teaching, and motivate teachers to seek excellence in teaching.^7^ Our university encourages all the faculty to learn PBL teaching, sets standards in the operation and sets funds to honor the faculty who will contribute to the teaching before adopting the teaching method.

### Prepare students for their role and responsibility

PBL is a learning process that requires students to be actively involved in collaborative group work. Students are the main characters in the process so they should understand their roles in PBL tutorials. However, in practical teaching, students only have a hazy idea of what PBL entails, even though most schools using PBL as a main element of their program are very explicit about what it entails.^8^ There is a need for training them in workshops on how to learn and what to do in this curriculum. Our university provides a lesson about what is best for students to do in this system. For example, the lesson require student to search for literature, do presentations, raise questions and make discussions on their own before teachers organize the discussions. Such training will enable them with skills of literature searching, preparing their self-directed learning issues, and understanding their roles in PBL tutorials.^6^

### Carefully select and present clinical and imaging sources

Preparation of clinical and imaging data is prerequisite for teaching. They deserve careful selection and presentation. Teaching staff should choose typical cases for teaching. In our university, teachers are also doctors. After obtaining written agreements from patients, the teacher collects their clinical data, including basic information, chief complaint, symptoms, signs, and laboratory examinations based on the syllabus. Students are requested to sign the consent form before the class to avoid any privacy disclosure. The data is presented in words or pictures (some identical or significant signs). Imaging data and surgery materials are presented in images and videos, respectively. Select materials suiting different stage of students according to their current knowledge and teaching requirement curriculum, and provide students with the opportunity to encourage their research skills and lateral thinking.^9^

### Promote clinical thinking and interpersonal skills of students

Clinical thinking is defined as centering on a patient’s narrative with history, physical examination and laboratory data, to summarize the characteristic and extract meaningful concepts leading to diagnosis and management. Content analysis is applied in PBL teaching in our university to make important contributions to improving clinical thinking skills for medical students.^10^ That includes measurements of the length of speaking, participate rates, social clues, interactions and speech of students in class. What’s more, debate, a time-honoured method of cultivating the active engagement of students, is also applied to our teaching.^11^ It not only encourages the critical analysis^12^ but also develops interpersonal skills. In this way, they will learn how to respect each other and debate in a proper way while delivering their contradictory perspectives.

### Provide student and teaching staff with feedback

To improve the work efficiency, feedback from students and staff was introduced in our school. (1)Feedback from teaching staff: the tutors are given an opportunity to deliver feedback on the case and challenges they faced with their groups and assess the structure, and design of the curriculum and areas that need improvement and enhancement. The feedback can be provided in verbal or written or even through the WeChat anytime. Besides, workshops are a useful mechanism and can be scheduled at a convenient time for faculty feedback. It emphasizes the application of adult learning theories to on-the-job teaching strategies and provides opportunities for peer discussions and skills practice.^13^ (2) Feedback from students: Clinical teachers can improve their skills and teaching efficiency from the feedback from students. Feedback can ensure the flow of the curriculum meets the learning needs of the students and make a successful implementation to the curriculum.^6^ Students might provide their views about anything, like the study contents, suggestions for presentation, through electronic questionnaires, voice, letters or even in class. In our university, a regular questionnaire in WeChat is given every two months to teachers and students.

### Establish a new curriculum committee

To standardize and unify the teaching process, it is necessary to establish a new curriculum committee. The committee works with the department of medical education to establish working groups and subcommittees needed for the construction and implementation of the new curriculum. Specific duties and responsibilities should be formulated^14^ including which disciplines should be involved in the design per course, which learning tasks are covered and which disciplines are involved in a specific course.

## Conclusions

In an era of rapid advances in new media, traditional PBL teaching method can’t satisfy teaching requirements. While there are many online tools for teaching and learning which are introduced into the PBL teaching,^15^ the WeChat-PBL teaching wins because of its popularity in China. Use of WeChat is not limited to those who have access to a computer, and users can communicate from any device with internet capabilities. It breaks the spatial and temporal limitations of the traditional classroom, stimulates students’ learning enthusiasm, improves their practical abilities, and enhances their sense of teamwork. The strategies we put forward above are intended to maximize its effects. However, more strategies still need exploration.

### Acknowledgements

This study was supported by Education and Teaching Reform Research Project of Central South University (No. 2016jy88) and Hospital Management Research Project of Xiangya hospital, Central South University (No. 016GL18).

### Conflicts of Interest

The authors declare that they have no conflict of interest.
